# Incorporation of *Lactococcus lactis* and Chia Mucilage for Improving the Physical and Biological Properties of Gelatin-Based Coating: Application for Strawberry Preservation

**DOI:** 10.3390/foods13071102

**Published:** 2024-04-03

**Authors:** Mingrui Li, Zhikun Yang, Xiaodong Zhai, Zhihua Li, Xiaowei Huang, Jiyong Shi, Xiaobo Zou, Guanhua Lv

**Affiliations:** 1 Agricultural Product Processing and Storage Lab, School of Food and Biological Engineering, Jiangsu University, Zhenjiang 212013, China; 2222118071@stmail.ujs.edu.cn (M.L.); yangzhikun22ujs@163.com (Z.Y.); zhai_xiaodong@ujs.edu.cn (X.Z.); lizh@ujs.edu.cn (Z.L.); huangxiaowei@ujs.edu.cn (X.H.); 2212118055@stmail.ujs.edu.cn (G.L.); 2 Institute of Future Food Technology, JITRI, Yixing 214200, China; 3 International Joint Research Laboratory of Intelligent Agriculture and Agri-Products Processing, Jiangsu University, Zhenjiang 212013, China

**Keywords:** chia mucilage, *Lactococcus lactis*, strawberry, gelatin

## Abstract

In this work, a gelatin/chia mucilage (GN/CM) composite coating material doped with *Lactococcus lactis* (LS) was developed for strawberry preservation applications. The results of the scanning electron microscope and Fourier transform infrared spectroscopy stated that the enhanced molecular interaction between the CM and GN matrix strengthened the density and compactness of the GN film. Antifungal results indicated that the addition of LS significantly (*p* < 0.05) improved the ability of the GN coating to inhibit the growth of *Botrytis cinerea* (inhibition percentage = 62.0 ± 4.6%). Adding CM significantly (*p* < 0.05) decreased the water vapour permeability and oxygen permeability of the GN coating by 32.7 ± 4.0% and 15.76 ± 1.89%, respectively. In addition, the incorporated CM also significantly (*p* < 0.05) improved the LS viability and elongation at break of the film by 13.11 ± 2.05% and 42.58 ± 1.21%, respectively. The GN/CM/LS composite coating material also exhibited an excellent washability. The results of this study indicated that the developed GN/CM/LS coating could be used as a novel active material for strawberry preservation.

## 1. Introduction

Strawberries (*Fragaria × ananassa*) are among the most popular fruits worldwide because of their pleasant taste, aroma, and colour. Regular consumption of strawberries can help to maintain normal levels of vitamins and various trace elements in the body. However, strawberries are susceptible to water loss and infection with moulds such as *Botrytis cinerea* because of their loose texture and high sugar content, thus causing compromised nutritional value and sensory properties [1]. Therefore, suitable preservation methods are necessary to preserve the value of strawberries.

Coating strawberries is a common method of preservation. In particular, coatings prepared from novel materials that are safe, eco-friendly, and economical can reduce the adverse impacts of petroleum-based plastic materials on human health and the environment [2,3]. The rates of water loss, matter exchange, and metabolism of coated strawberries are slowed down, thereby slowing down quality loss and ageing deterioration [4]. Different coating materials derived from natural biological macromolecules (e.g., lipids, polysaccharides, and proteins) have been widely explored because of their biodegradability and biocompatibility. Gelatin (GN), a polypeptide chain produced by acid or alkaline hydrolysis of collagen, is widely used as a preservation material due to its non-toxicity and superior ability to produce transparent coatings [5,6].

However, the resistance to *B. cinerea* of the GN coating is insufficient to effectively preserve strawberries [7]. Many studies have been conducted to enhance the antimicrobial activity of GN coatings by adding flavonoids, metal nanoparticles, and probiotics [8]. Probiotics are a group of microorganisms that, when ingested in sufficient quantities, can improve host immunity and intestinal flora balance. In addition, probiotics can also inhibit the growth of harmful microorganisms by releasing antimicrobial metabolites [9,10]. Various probiotics have been incorporated into coating materials to enhance antimicrobial activity, such as *Lactiplantibacillus plantarum* 299V, *Lacticaseibacillus casei* 01, and *Lactobacillus salivarius* NRRL B-30514 [11,12,13]. Among them, *Lactococcus lactis*, a lactic acid bacteria strain, is recognised worldwide as a safe microorganism with probiotic functions [11]. *L. lactis* can inhibit fungal growth by producing antifungal compounds such as and diacetyl [14], as well as by synergistic effects of fermentation products such as organic acids. Recently, *L. lactis* isolated from the food industry significantly inhibited mould activity [15]. Gajbhiye et al. [16] isolated and purified antifungal compounds such as cyclo-dipeptide from *L. lactis*, demonstrating the potential of *L. lactis* to be applied to fungal control in food preservation. Navale et al. [17] found that *L. lactis* collected from milk showed effective inhibition of fungi because of the presence of metabolites such as phenolic compounds. Hence, considering the probiotic function and potential antifungal activity of *L*. *lactis*, it was expected to be incorporated as an ideal bioactive substance to enhance the antifungal properties of the GN coating.

Although adding bioactive substances can enhance the biological properties of coatings, the barrier and mechanical properties of GN coating are still insufficient. In addition, the biological properties of probiotic films are closely related to the viability of probiotics. The limited probiotic protection ability of pure GN will cause a reduction in the biological activity of probiotic films.

Chia mucilage (CM), a high-molecular-weight polysaccharide derived from chia seeds, is mainly composed of α-D-glucose, β-D-xylose, and 4-O-methyl-α-D-glucuronic acid [18]. Various works [19,20,21] have demonstrated that CM could enhance the mechanical and barrier properties of protein-based films by forming cross-linked complex network structures by protein–polysaccharide interactions, as well as through the hindrance of moisture and gas movement by the intertwined CM polysaccharide chains. Luo et al. [19] found that incorporating CM into the GN matrix reduced the water vapour permeability (*WVP*) and increased the elongation at break (*EAB*) of the material. In addition, CM has been used as a protective agent for probiotics by providing a physical barrier and nutrients due to its dense polysaccharide network and high amount of dietary fibre [22,23]. 

However, there are few studies on CM to improve *L. lactis* viability in coatings and coating properties. Hence, the present research aimed to add *L. lactis* and CM into the GN matrix to fabricate a novel active coating material for strawberry preservation. We investigated the effect of CM and *L. lactis* on the water resistance, gas barrier, and mechanical and biological characteristics of the active coating materials. The microstructure, molecular interaction, and *L. lactis* viability in the coating were also assessed. Finally, the potential preservation effect of the active coating material on the strawberry postharvest quality was measured by analysing the weight loss, firmness, total soluble solids (TSS), and decay index at 25 ± 1 °C. This work may open a new approach to improving the quality of strawberries and reducing agricultural waste and economic losses.

## 2. Materials and Methods

### 2.1. Materials

*L. lactis* (CICC 20090) and *B. cinerea* (AS3. 3789) were sourced from the China Centre of Industrial Culture Collection (Beijing, China) and Shanghai Biology Collection Centre (Shanghai, China), respectively. Gelatin (CAS 9000-70-8, CP) was purchased from Shhushi (Shanghai, China). Chia seeds (CS, each 100 g contained 1814 kJ of energy, 24.9 g of protein, 30.0 g of fat, 1.5 g of carbohydrate, and 31.9 g of dietary fibre) and strawberries were purchased from local suppliers. All other chemicals utilised in this study were procured from Nanjing Jiancheng Technology Co., Ltd. (Nanjing, China). 

### 2.2. Preparation of Microorganisms

*B. cinerea* was inoculated onto plates of potato dextrose agar (PDA) and left to incubate within an incubator for 7 days at a temperature of 26 °C. After this incubation period, sterile distilled water was carefully poured over the plates and the spores were dislodged by gently rubbing the plates with a sterile blade. To prevent the presence of any mycelial debris, the spore suspensions were passed through four layers of sterile gauze. The concentration of spores was then adjusted to 1 × 10^6^ spores/mL using a haemocytometer for future use.

*L. lactis* was incorporated into the liquid MRS (Man, Rogosa, and Sharpe) medium and incubated in a shaker at 30 °C and 160 rpm for a day. In total, 2 mL of this culture was then transferred to 100 mL of fresh MRS and kept in the same conditions. After incubation, the culture was centrifuged at 5000 rpm for 10 min at 4 °C, with the supernatant being discarded. The sedimentation was rinsed twice with sterile water before being re-suspended in 4 mL of sterile water to create the *L. lactis* suspensions. Furthermore, pre-experiments revealed that too low a concentration did not satisfy the coating requirements (>7 log_10_ CFU/g), and too high a concentration resulted in low survival rates, wasting resources. Hence, the concentration of the suspension was adjusted to 1 × 10^10^ cells/mL using a haemocytometer.

### 2.3. CM Extraction

CM was extracted from CS according to the method of Luo et al. [19]. Firstly, CS and distilled water were mixed in a ratio of 1:30 (*w*/*v*) and stirred at 50 °C for 3 h. After that, the solution was treated using sonication (KH-400DS, Kunshan Hechuang Ultrasonic Instruments Co., Ltd., Kunshan, China) for 2 min and centrifuged at 10,000 rpm for 30 min at 4 °C. Finally, the CM was filtered through a filter cloth and collected and stored at 4 °C.

### 2.4. Preparation of Coatings Materials

The previous method [24] was slightly modified to prepare the coating material. The GN coating solution (2.0%, *w*/*v*) was created by solubilizing the GN powder in distilled water at 60 °C in a water bath. To create the GN/CM coating solution, 30 mL of the prepared CM was added to the GN solution, and the solution was stirred in a water bath at 60 °C for 45 min until the coating components were thoroughly mixed. Subsequently, the GN/CM solution was cooled to 30 °C and de-aerated via ultrasound for 30 min to exhaust air. Pre-experimental results showed that the antifungal activity of the film could not be obviously improved when the addition concentration of *L. lactis* was lower than 1%, and when the addition concentration of *L. lactis* was higher than 4%, it negatively affected the mechanical properties and the viability of probiotics in the film. Therefore, the ideal concentration range of the probiotic solution is 1–4%. Different contents (1%, 2%, 4% *v*/*v*) of *L. lactis* suspension were incorporated into the GN/CM mixture to produce GN/CM/1.0% *L. lactis* (GN/CM/LS1), GN/CM/2.0% *L. lactis* (GN/CM/LS2), and GN/CM/4.0% *L. lactis* (GN/CM/LS4) coating solutions, respectively. To study the physicochemical properties of the coatings, the coating solutions mentioned above were poured onto plates and dried in an oven set at 30 °C for 24 h to obtain different films. Formed films were peeled off and stowed in zipped bags at 25 °C for further analysis.

### 2.5. Viability of L. lactis in Coatings

As a control for the cell viability assay, 1%, 2%, and 4% (*v*/*v*) *L. lactis* were added to the GN coating solution to prepare GN/1.0% *L. lactis* (GN/LS1), GN/2.0% *L. lactis* (GN/LS1), and GN/4.0% *L. lactis* (GN/LS1) coating solutions and film samples. The average humidity in most parts of China is about 75%, so the bioactive film loaded with *L. lactis* was stored at a relative humidity (RH) of 75% and 25 ± 1 °C during the 24-day assay period. In accordance with the previous method, the vitality of the probiotic was evaluated every 4 d [24]. A total of 1 g of films was aseptically added to a sterile saline solution and stirred to ensure the release of all probiotics into the mixture. Then, the mixture was spread on the MRS agar medium, and the *L. lactis* was counted using the plate count method. Furthermore, cells in coating solutions were counted using the identical method before drying. To determine the survival rate, the following formula was used:

(1) $$Survival\;rate\left(\%\right)=N/N_{0}\times 100$$

The variables $N_{0}$ and N denote the quantity of initially viable cells in the film (measured in CFU/g) and the number of viable cells following a specific storage period, respectively.

### 2.6. Antifungal Capability

Although films prepared from aqueous solutions may release more antifungal compounds in the medium than upon application, based on previous work [25], we selected to employ the method of covering the plate with films to compare the antifungal activity against *B. cinerea* of the various film groups. For inoculation, 15 µL of *B. cinerea* spores was point-deposited at the centre of the PDA medium (9 cm diameter). After that, GN-based films previously prepared were set on the PDA plate inoculated with *B. cinerea* spores. The *B. cinerea* inoculated PDA plate without film treatment served as a control. All treatments were repeated in triplicate. 

Radial extension of the colony in all samples was determined when the *B. cinerea* grown on the control completely covered the plate. The calculation of radial inhibition percentage was carried out by implementing Equation (2):

(2) $$\mathrm{Antifungal}\;in\mathrm{dex}\;(\%)\;=\left(\left(X_{c}-X_{i}\right)/X_{c}\right)\times 100$$

$X_{i}$ and $X_{c}$ mean the radial growth (mm) of fungus in the treated and control plates, respectively.

### 2.7. Oxygen Permeability (OP)

The gas barrier property of the coating was assessed by measuring *OP* following the previous approach [26]. The coating material was tested using an automated *OP* testing machine, and the *OP* values were calculated by Equation (3).

(3) $$OP=\left(OTR\times x\right)/∆P$$

where x denotes the oxygen partial pressure, ∆P represents the thickness of the film sample, and OTR denotes the oxygen transmission rate.

It should be noted that according to the ASTM standard, the test was performed under an almost 0% humidity condition. The humidity environment in the application may cause deviations in the *OP* values.

### 2.8. Scanning Electron Microscope (SEM)

The cross-sectional morphology of the coating materials was obtained using SEM (JSM-7800F, JEOL, Musashino City, Japan). The samples were attached to an aluminium stub with electrically conductive double-sided tape, plated with gold using a sputtering process, and then observed with a 15 kV accelerating voltage and a 400–4000 fold magnification.

### 2.9. Fourier Transform Infrared (FTIR) Spectroscopy

The FTIR spectra of the coatings were obtained using the Nicolet iS50 FTIR spectrometer (Thermo Fisher Scientific, Waltham, MA, USA) with a resolution of 4 cm^−1^ across 16 scans in attenuated total reflectance mode (ATR) utilising a platinum crystal accessory for a band of 600–4000 cm^−1^. A background spectrum of an open beam was recorded. Furthermore, each measurement was carried out three times.

### 2.10. Mechanical Performance

The *EAB* and tensile strength (*TS*) of samples were assessed using the Instron Universal Testing Machine (Model 4500, Instron Corporation, Canton, MA, USA) with reference to the ASTM D882-00 standard procedure with little modification. Coating materials were placed between the tension and the initial grasp after being cut into 6.0 cm × 2.0 cm strips. Tests were then performed utilising an initial grip distance of 40 mm and a crosshead speed of 1.0 mm/s.

### 2.11. Colour Properties 

Coatings were subjected to measurements of the colour parameters L* (lightness/brightness), a* (redness/greenness), and b* (yellowness/blueness) with a CR-400 Minolta colourimeter (Minolta Camera, Co., Ltd., Osaka, Japan). The colour indicators (L*, a*, and b*) were identified by the mean value of three randomly chosen spots on the film sample.

### 2.12. Moisture Content (MC) 

The *MC* of samples was determined by a Halogen Moisture Analyzer (VM-E, VICOMETER, Taizhou, China).

### 2.13. Water Vapour Barrier Property

The *WVP* of the produced coating material was evaluated with reference to ASTM technology (ASTM Standard E96M-05). Specifically, centrifuge tubes filled with distilled water and sealed with film were placed in a desiccator at 50% RH and 25 °C. The weight of tubes was measured hourly until a steady rise in weight was observed. The water vapour transmission rate (*WVTR*) of the coating material was first calculated from Equation (4) and then the *WVP* from Equation (5):

(4) WVTR=∆m/(A×∆t)

(5) WVP=WVTR×(x/∆P)

where ∆m/∆t represents the change in water mass during the unit transfer time; A represents the film area through which water is transferred; ∆P is the difference in partial pressure of water vapour on both sides of the film; and x is the thickness of the film.

### 2.14. Coating Washability

The washability of the GN-based coatings was assessed by dissolution in water. A film slice was placed in a glass vial containing distilled water at room temperature and gently shaken. The dissolution of the film was timed and photographed to record the process for analysis. 

### 2.15. Application of Coatings in Preserving the Strawberry

**Scheme 1:**
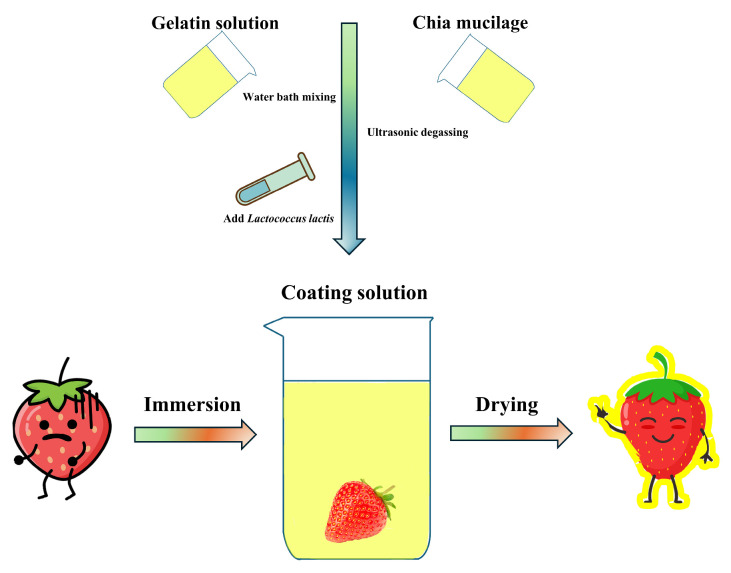
Preparation of coating solutions and the process of coating strawberries.

Strawberries are seasonal fruits, and their quality mainly depends on the timing of harvesting. Therefore, for preservation experiments, the strawberries were purchased within a short span of a few days. Strawberries were carefully washed with tap water and put at 25 ± 1 °C to dry. Then, the strawberries were immersed in the different coating solutions, subsequently removed, and left at 25 ± 1 °C until dry (Scheme 1). Strawberries without coating served as a control. Finally, we stored all the samples in polypropylene packages (a top diameter of 17.3 cm, a bottom diameter of 12.8 cm, and a height of 8 cm) with lids and vents at 25 ± 1 °C and 75% RH for 6 days and carried out relevant measurements in each period.

#### 2.15.1. Weight Loss Rate

The weight loss of strawberries was measured by recording the weights of polyethene boxes containing the strawberries at each storage period, using Equation (6):

(6) $$Weight\;loss={(W}_{0}-W_{1})/W_{0}\times 100\%$$

where $W_{0}$ represents the initial weight, and $W_{1}$ denotes the final weight of each measurement period.

#### 2.15.2. Firmness Determination

The firmness of strawberries was determined by employing a texture analyser (TA-XT Plus, Stable Micro Systems, Godalming, UK). Strawberries were penetrated by a P/5 cylindrical probe at 1.0 mm/s to 5 mm depth, while their firmness was expressed in Newtons (N).

#### 2.15.3. TSS

After homogenising strawberries (5 g), the filtrate for the test was collected, and the TSS concentration in strawberries was determined by a brix meter (ATAGO RX-5000a, ATAGO Company, Tokyo, Japan).

#### 2.15.4. Decay Rate

Strawberry spoilage was assessed by analysing the area of spoilage of each strawberry. The assessment was made using the following scale: where 0 indicates no decay; 1 indicates a decayed area of less than 20% of the strawberry area; 2 indicates a decayed area of 20–50% of the strawberry area; 3 indicates a decayed area of more than 50% of the strawberry area. The strawberry decay rate was obtained using the following formula.

(7) $$Decay\;rate\left(\%\right)=\sum \left(Decay\;level\times The\;number\;of\;strawberries\;in\;this\;level\right)/(3\times n)$$

where n represents the number of strawberries rated 3.

### 2.16. Statistical Analysis

Minitab statistical software version 17 (Minitab Inc., Lock Haven, PA, USA) was run to analyse significant differences (*p* < 0.05) using a one-way analysis of variance (ANOVA) and Tukey’s test. Three batches of films were prepared under the same conditions at different times and each experiment was tested three times for technical replication.

## 3. Results

### 3.1. Viability of L. lactis in the Coating

**Figure 1:**
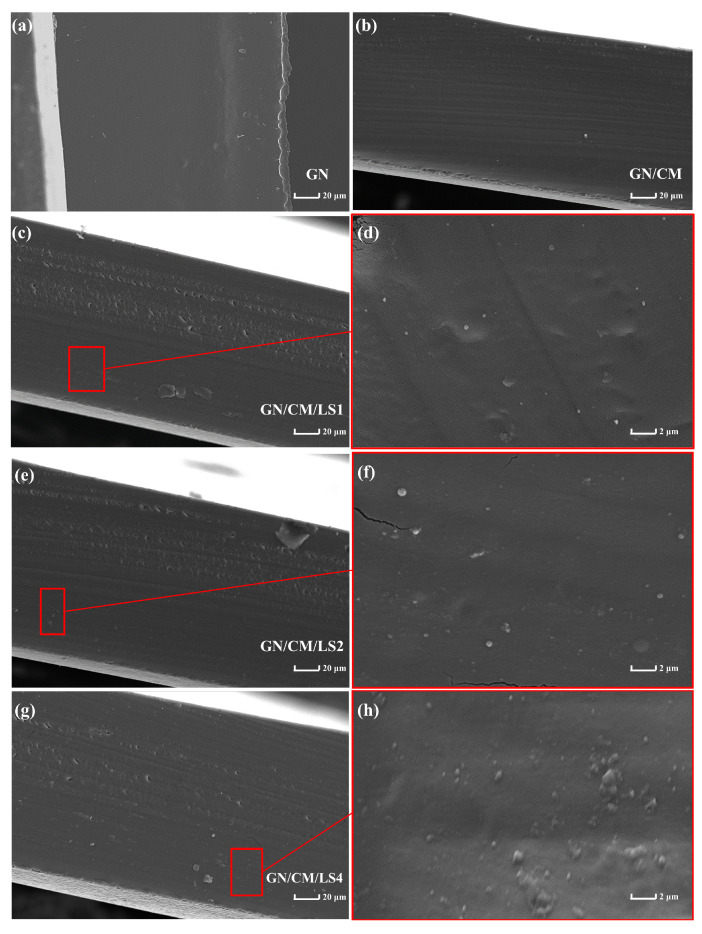
Cross-sectional scanning electron microscope images of GN (**a**), GN/CM (**b**), GN/CM/LS1 (**c**), GN/CM/LS2 (**e**), and GN/CM/LS4 (**g**) films and magnified images of probiotic films (**d**,**f**,**h**). GN: gelatin; CM: chia mucilage; LS: *Lactococcus lactis*.

**Figure 2:**
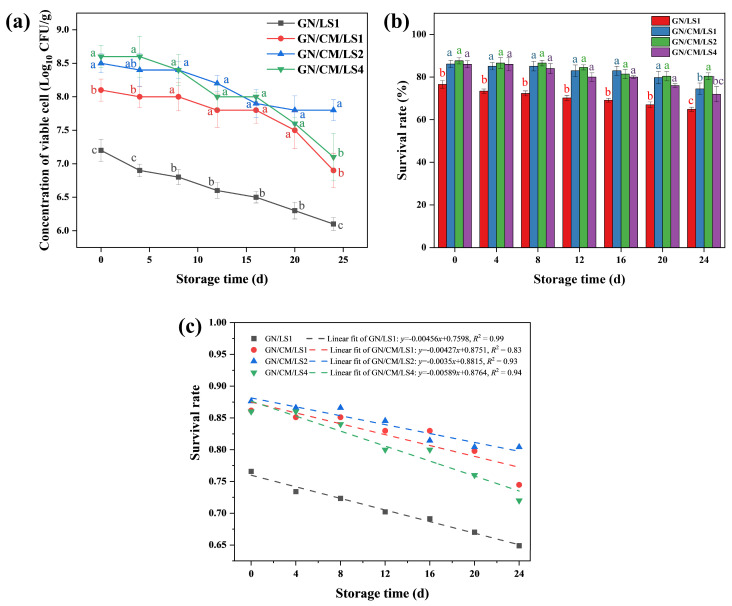
The number of viable cells (**a**), cell survival (**b**), and a linear fit curve for survival (**c**) of *Lactococcus lactis* in films. Non-significant (*p* > 0.05) and significant (*p* < 0.05) differences are indicated by the same and different letters, respectively.

Ensuring microbial survival during coating processing and application is crucial in developing bioactive coatings. The drying process generated heat and osmotic stress, which could be harmful to *L. lactis*. A protein or carbohydrate matrix such as GN and CM could protect the structural integrity of cells as a protective barrier by enclosing the cells during the drying process [27]. This explanation corresponds to the SEM images shown in Figure 1d,f,h, which show that *L. lactis* cells with complete structure were fully embedded in the GN/CM matrix. Notably, during the drying process, the GN/CM/LS1 group had a significantly (*p* < 0.05) higher survival rate (Figure 2b; Day 0) than the GN/LS1, indicating that CM was effective in reducing damage to LS. The cross structure formed by the CM and GN better encapsulated the cells and reduced heat and water evaporation shocks.

Figure 2a,b show the viable cell number and the survival rate of *L. lactis* in the coatings within the storage period, respectively. The microorganism viability in biopolymer coatings depends on several aspects, including the initial concentration of microbes, accumulation of metabolism, cohesive bonds between the coating matrix and the cells, and the nutrients required by the cells [28]. As depicted in Figure 2a,b, the cell number (7.8 ± 0.16 Log CFU/g) and survival rate (80.41 ± 1.60%) of *L. lactis* in GN/CM/LS2 were significantly (*p* < 0.05) the highest after 24-day storage. The growth competition and the large number of harmful metabolites produced by the high concentration of probiotics in the GN/CM/LS4 might decrease the survival rate [29]. Kanmani et al. [30], who added *Lactobacillus rhamnosus* GG, *L. reuteri,* and *L. acidophilus* into pullulan/starch edible films, similarly found that the accumulation of metabolites might inhibit cell growth. The number of cells and survival were significantly lower in GN/LS1 than in GN/CM/LS1 during storage (*p* < 0.05). The same trend occurred between GN/LS2 and GN/CM/LS2, and between GN/LS4 and GN/CM/LS4 (Figure S1). The absence of CM prevented the probiotic cells from obtaining enough nutrients to maintain cell viability. Oliveira-Alcântara et al. [31], who researched bacterial cellulose/cashew gum edible films mixed with *Bacillus coagulans*, and Soukoulis et al. [32], who explored the stability of *L*. *rhamnosus* GG in films, also suggested that the nutrients could elevate cell viability in the films. In addition, a first-order kinetic fit curve for survival (Figure 2c) was plotted to facilitate observation of the trend of probiotic cell inactivation in the coating material. It could be observed that the curve of GN/LS1 was located at the bottom (lowest survival rate), while the curve of GN/CM/LS4 had the largest absolute value of slope (−0.00589, fastest decline in survival rate). The curve of GN/CM/LS2 was located at the top and had the smallest absolute value of slope (−0.0035, the highest survival and slowest decline in survival), indicating that the GN/CM/LS2 formulation had the best capacity to maintain cell viability. 

### 3.2. Antifungal Property

**Figure 3:**
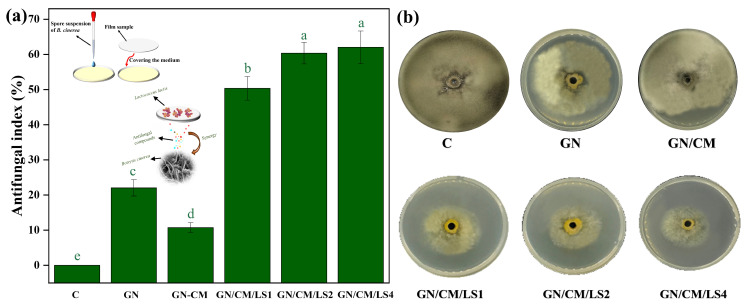
Antifungal index of films against *B. cinerea* (**a**) and a photograph (**b**) of *B. cinerea* treated with different films. Non-significant (*p* > 0.05) and significant (*p* < 0.05) differences are indicated by the same and different letters, respectively. C: control group.

The *B. cinerea* in the control group reached the maximum diameter of the plate on Day 7 and the samples were subsequently photographed and measured. The result of the bioactivity assessments of the GN-based coating material against *B. cinerea* is shown in Figure 3a,b. It was observed that all treatments had a positive radial inhibition, which meant a positive antifungal activity. The ability of the *L. lactis*-loaded films against *B. cinerea* was significantly improved compared to the other films (*p* < 0.05). This finding could be because of the antifungal chemicals in the exometabolome of *L. lactis* strains that effectively suppressed *B. cinerea*. Increasing the level of *L. lactis* from 1% to 2% significantly increased the antifungal ability of the film (*p* < 0.05). However, continuing to 4% did not significantly (*p* > 0.05) increase the antifungal ability. This might be due to the uneven release of antifungal substances caused by aggregation, which affects the bioactivity of the film.

Furthermore, it was found that GN and GN/CM coating materials also exhibited radial inhibition against *B. cinerea*. This result might be due to the coating having a barrier effect on the oxygen in the surroundings, leading to a reduction in the oxygen concentration underneath the coating [25]. *B. cinerea* is aerobic, and the decrease in oxygen concentration inhibited its growth. It was also observed that the antifungal activity of the GN/CM group was lower than that of the GN group, which might be due to the fact that *B. cinerea* utilises the nutrients from the CM.

### 3.3. FTIR Analysis and SEM Micrographs

**Figure 4:**
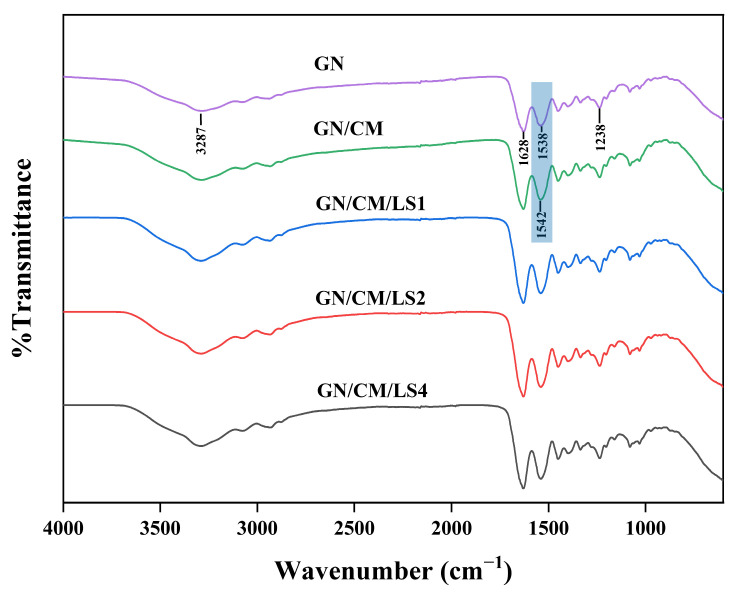
Fourier transform infrared spectra of GN, GN/CM, GN/CM/LS1, GN/CM/LS2, and GN/CM/LS4 films.

The functional groups and intermolecular interactions of coatings could be studied by FTIR spectral analysis (Figure 4). The GN film pattern showed that the principal absorption bands were N-H stretching at 3287 cm^−1^ (amide A), C=O stretching at 1628 cm^−1^ (amide I), N-H bending at 1538 cm^−1^ (amide II), and C-N stretching at 1238 cm^−1^ (amide III) [33]. The shift in the amide II (from 1538 cm−1 of GN to 1542 cm^−1^ of GN/CM) band indicated a successful interaction between CM and GN. The CM polysaccharides contained sufficient hydroxyl and carboxyl groups, which could form hydrogen bonds with the -NH-groups of the GN proteins. These hydrogen bonds led to the shift of amide II [19].

In addition, there was an enhancement of the bands near 3287 cm^−1^ after the addition of *L*. *lactis*, which was attributed to the presence of hydroxyl groups in *L*. *lactis*. This result was similar to the study of Yang et al. [24] whose FTIR results showed an increase in intensity in the 3000–3667 cm^−1^ range corresponding to the hydroxyl group after the addition of *Saccharomyces cerevisiae* cells. 

SEM was utilised to evaluate the visualisation of probiotic cells and the micromorphology of biopolymer coatings. The cross-sectional pictures of the coating materials are displayed in Figure 1. The pure GN film had a homogeneous and smooth structure. The addition of CM made the GN film dense and compact. This was in accordance with the fact that the cross-linking network was established within the polymer matrix of films by the enhanced molecular interactions between GN and CM (Figure 4). The GN/CM film became rough after the addition of probiotics, and the roughness of the film was increased with the increasing content of the probiotic. Ebrahimi et al. [34] also discovered that the cross-section of the carboxymethyl cellulose films became rough after the incorporation of *Lactobacillus acidophilus*, *L. casei*, *L. rhamnosus*, and *Bifidobacterium bifidum*. This was related to the aggregation of probiotic cells in the film matrix. This result indicated that the GN/CM-based probiotic carrier film was prepared successfully in the present work.

### 3.4. Mechanical Properties

Qualified coating material generally possesses strong mechanical characteristics to handle storage and transportation pressures. According to Amiri et al. [35], the *TS* is associated with the coating resistance to tension forces (preventing cracking and breakage), whereas the *EAB* is related to the capacity for stretching and flexibility (accommodating the natural expansion and contraction of the food). The *EAB* of the GN/CM film significantly (*p* < 0.05) elevated to 39.167 ± 0.643%, 42.58% higher than the GN (Table 1). This result might be accounted for by CM acting as a plasticizer in GN films and was identical to the results of Luo et al. [19], who discovered that the *EAB* in GN films significantly increased with adding CM. Parallel findings by Orozco-Parra et al. [36] and other researchers [37,38] also revealed that various carbohydrates, such as polysaccharides, glucose, and fructooligosaccharides, showed a plasticization in the films, reducing *TS* while enhancing *EAB*. Moreover, when *L. lactis* was added to the composite films, there was a slight (*p* > 0.05) rise in *EAB*. This result might be because of the interfacial interaction between the matrix and cells [39]. In contrast, Salimiraad et al. [40] found that introducing microorganisms into a film caused a reduction in *TS* and *EAB*. This reduction happened because the microorganisms damaged the adhesive connections of the film structure. Table 1 shows that the GN/CM films had a significantly (*p* < 0.05) lower *TS* than GN. This finding corresponded to those mentioned above that CM polysaccharides could act as plasticizers, increasing the *EAB* of the film but decreasing the *TS*. The reduction in *TS* and increment in *EAB* might be due to the strengthened effects on the film matrix via interactions between protein and polysaccharide chains. Tymczewska et al. [41] also obtained similar results. In their experiments, the *TS* of the films decreased with the addition of a plasticizer (glucose and glycerol). Incorporating *L. lactis* continued to decrease the *TS*. As mentioned above, *L. lactis* weakened the adhesive bonds in the coating matrix structure, thus reducing the *TS*. The same trend was found by Piermaria et al. [42]. They found that less force was needed to break the biomacromolecule film because the microbe could cause discontinuities in the matrix. Nevertheless, there was no significant (*p* > 0.05) change in *TS* for the GN/CM/LS2 and GN/CM/LS1 films, except for GN/CM/LS4. The reduction in *TS* in GN/CM/LS4 might be related to the fact that a high concentration of *L. lactis* could aggregate in the matrix, thus reducing the compactness of the GN/CM matrix (Figure 1) [39]. 

### 3.5. Gas Barrier Property

**Figure 5:**
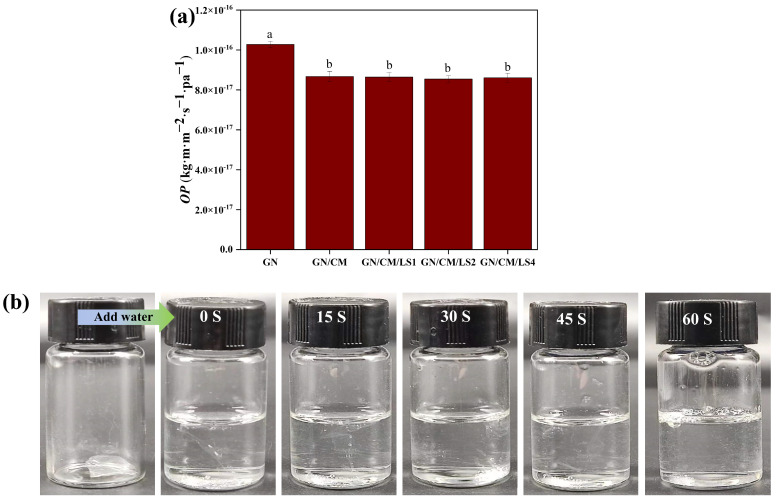
The oxygen permeability (*OP*; (**a**)) and photographs of rapidly dissolving in distilled water (**b**) of the film samples. Non-significant (*p* > 0.05) and significant (*p* < 0.05) differences are indicated by the same and different letters, respectively.

Controlling the *OP* of the coating is beneficial to reducing the respiration rate of agricultural products as it reduces the contact of the food with oxygen. The slowed respiration rate reduces metabolic and oxidative losses of nutrients, thus extending the shelf-life. After adding CM, the *OP* of the GN coating materials reduced significantly (*p* < 0.05) from (10.27 ± 0.16) × 10^−17^ kg·m·m^−2^·s^−1^·pa^−1^ to (8.67 ± 0.26) × 10^−17^ kg·m·m^−2^·s^−1^·pa^−1^ (Figure 5a). As shown in Figure 1a,b, the decrease in *OP* could be attributed to the fact that incorporating CM tended to tighten the structure of the GN films, which impeded the passage of oxygen. Liu et al. [43] also suggested that incorporating crosslinkers formed complex pathways in the films, reducing *OP*. 

### 3.6. MC and WVP

*MC* is associated with the overall volume of water molecules within the coating network microstructure and is essential for sustaining the viability of *L. lactis* [44]. The *MC* value significantly decreased from 15.783 ± 0.294% (GN) to 13.533 ± 0.356% (GN/CM) with adding CM (*p* < 0.05) (Table 1). The result might be explained by the polysaccharide occupying hydrophilic protein sites, making these sites less accessible to water molecules. Javadian et al. [45] also found the same trend that ribose reduced *MC* in fish GN films. However, adding *L. lactis* or increasing its concentration significantly (*p* < 0.05) elevated the *MC* of the GN/CM film since *L. lactis* contained a substantial number of hydrophilic substances. 

The barrier property of coating material between food and atmospheric moisture is represented by *WVP*. As displayed in Table 1, the *WVP* of coating material significantly reduced from (7.017 ± 0.808) × 10^−13^ to (4.719 ± 0.533) × 10^−13^ kg·m·m^−2^·Pa^−1^·s^−1^ with adding CM (*p* < 0.05). Hazaveh et al. [46] suggested that the lower *WVP* resulted from matrix interaction (Section 3.3) since it decreased the free space of the film network. SEM images (Figure 1a,b) also show that CM made the films tighter. Moreover, incorporating *L. lactis* increased the *WVP*, and the *WVP* continued to increase with growing concentrations of *L. lactis*. According to SEM images, the increase in *WVP* might be due to the fact that *L. lactis* roughened the film, disrupting the uniform and dense structure of the film [34]. Fortunately, unlike 4.0%, adding 1% and 2% *L. lactis* did not significantly (*p* > 0.05) raise the *WVP* of the coating material. Coimbra et al. [47] also discovered that adding biologically active cultures into the film significantly increased the *WVP* since excessive cell concentration reduced the compactness and cohesion of the polymer matrix, thus favouring water penetration. In contrast, Orozco-Parra et al. [36] observed that including probiotic bacteria significantly reduced *WVP* compared to films with the same inulin concentration. This result could be because the polymeric matrix was disturbed by tiny microbial cell clusters, thus forming a complex pathway for water molecules to cross the polymer barrier. 

### 3.7. Washable Removal of the Coating

Some consumers value the washability of food coatings because of their personal eating habits. Based on the hydrophilic nature of proteins and polysaccharides, GN/CM-based coating materials could quickly swell or even dissolve when exposed to water. Figure 5b shows the dissolution process of GN-based films in distilled water. The film dissolved quickly within one minute, demonstrating excellent washability. Chang et al. [48] found that the bio-based coating was more accessible to wash in water and was safer, more environmentally friendly, and more convenient to consume than wax-based coatings.

### 3.8. Colour Properties 

Colour properties can affect the acceptability of coating. Therefore, we determined the colour characteristics of the coating materials, which are shown in Table 1. None of the measured parameters (L*, a*, b*, and Δ*E*) significantly (*p* > 0.05) differed among GN, GN/CM, GN/CM/LS1, GN/CM/LS2, and GN/CM/LS4. The outcome was the same as those of Sáez-Orviz et al. [49], who found that the appearances of control, probiotic, prebiotic, and synbiotic films were not significantly different in their experiment. 

### 3.9. Qualitative Assessment of Strawberries

The formulated films showed a satisfactory mechanical, barrier, and antifungal performance with potential for application to strawberries. We applied the coating materials to strawberries to further discuss the performance of the coatings in extending the shelf-life of strawberries.

#### 3.9.1. Appearance of Strawberries

**Figure 6:**
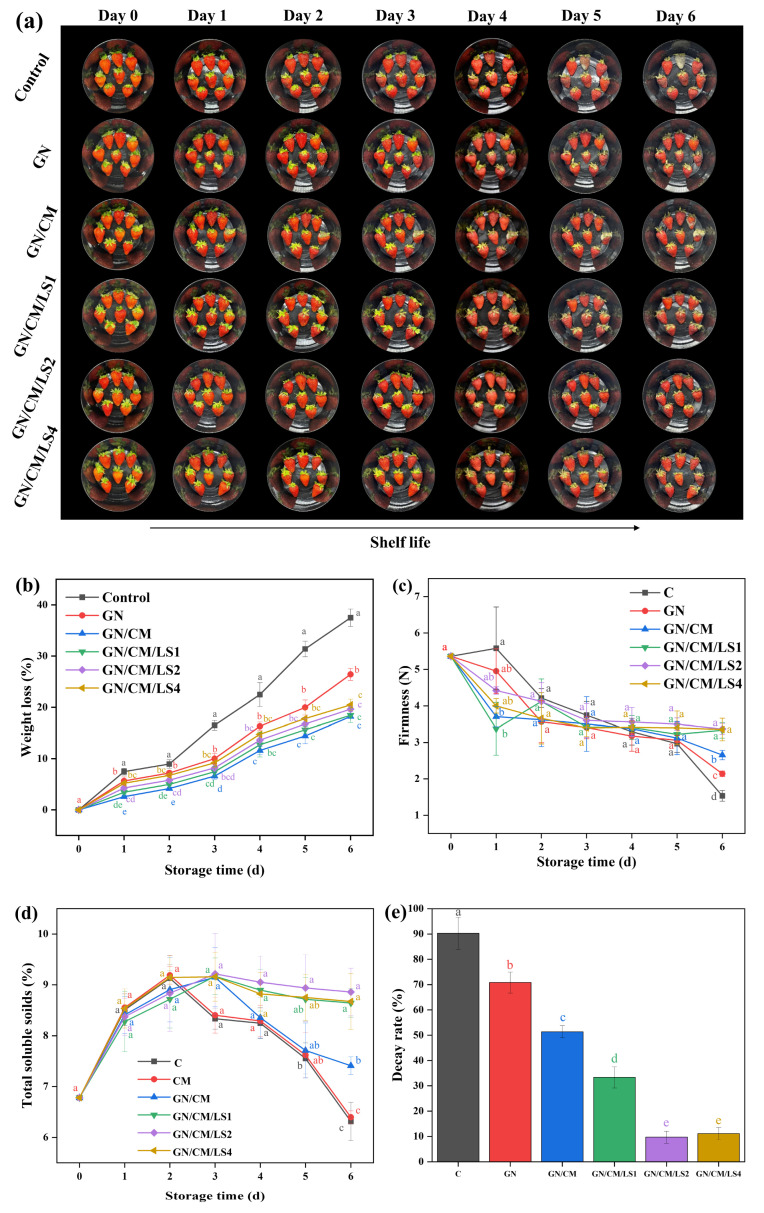
The appearance image (**a**), weight loss (**b**), firmness (**c**), total soluble solids (**d**), and decay rate (**e**) of strawberries coated with blank, GN, GN/CM, GN/CM/LS1, GN/CM/LS2, and GN/CM/LS4. Strawberries were kept at 25 ± 1 °C for 6 days. Non-significant (*p* > 0.05) and significant (*p* < 0.05) differences are indicated by the same and different letters, respectively.

Figure 6a depicts the appearance of strawberries during the six-day storage period. The strawberries initially had bright red surfaces and a low metamorphic rate. Over time, the strawberries appeared to have black spots and deteriorated. As expected, the blank group deteriorated more quickly and was even infected with *B. cinerea*. *B. cinerea* also grew on the strawberries treated with the GN/CM coating. This phenomenon corresponded to the in vitro antifungal assay results, where the GN/CM coating material showed the lowest antifungal ability of all samples. Furthermore, apparent rot was observed in strawberries treated with the GN coating.

The probiotic coating showed a better effect on preventing rotting and infestation with *B. cinerea* in strawberries. After six days of storage, the strawberries treated with *L. lactis*-rich coatings did not rot and were not infected with *B. cinerea*. 

#### 3.9.2. Weight Loss

During storage for six days, the weight of non-coated and coated strawberries progressively declined (Figure 6b). Coated strawberries containing different concentrations of *L*. *lactis* showed differences in weight loss during the first three days, which might be due to the different *WVP* and antifungal indexes of these coatings. The main reasons for weight loss are carbon loss through respiration and water evaporation through transpiration [50]. Microbial degradation of strawberry nutrients and cell walls can also contribute to weight loss. The semi-permeable barrier formed by the coating could inhibit the transfer of water and gases, and LS was also effective in inhibiting mould activity. On the last day, the weight loss rate in the control (37.50 ± 2.06%) was significantly (*p* < 0.05) more than that of the coated groups. Adding CM significantly (*p* < 0.05) reduced the weight loss. This might be attributed to the fact that CM reduced the *WVP* and *OP* of the coating (Section 3.5 and Section 3.6). Al-Hilifi et al. [51] also found that the GN coating added with hyaluronic acid polysaccharides reduced the weight loss of strawberries, possibly due to the interaction between hyaluronic acid and GN to form an enhanced barrier. 

#### 3.9.3. Firmness

Figure 6c depicts the change in the firmness of strawberries for 6 days. Both treated and untreated strawberries showed a decrease in firmness. After a 6-day metabolic and decomposition softening process, on Day 6, uncoated strawberries had the lowest firmness (1.53 ± 0.15 N) among the five groups. The coated strawberries showed a significant (*p* < 0.05) rise in firmness compared to the non-coated group, proving that GN-based coatings were effective in preventing the softening of strawberries, thus improving their edible quality. The inclusion of CM and LS in the coating both resulted in a significant (*p* < 0.05) improvement in strawberry firmness. The causes of strawberry softening are complex and are mainly caused by metabolic activities such as respiration and the breakdown of cell walls and pectin substances during strawberry ripening [52]. Mould infection might also promote the strawberry softening process by breaking down tissues and nutrients. CM improved the oxygen and water barrier of GN coatings, slowing down respiration and thus improving firmness, while LS inhibited mould growth and reduced mould degradation, thus maintaining firmness. 

#### 3.9.4. TSS

The TSS values in all groups first elevated and then reduced (Figure 6d). The TSS of the non-coated and GN-treated strawberries peaked the fastest, while other groups peaked one day later. The rise in TSS at the beginning of storage might be due to the decomposition of insoluble polysaccharides into soluble monosaccharides during strawberry ripening [53]. Adding CM could enhance the barrier ability of the GN coating, slowing down the ripening rate of strawberries and thus delaying the peak. After reaching the peak, the TSS started to decline, probably due to the continuous metabolism and mould degradation of soluble solids [54]. On Day 6, the TSS of control and GN groups decreased to 6.31 ± 0.38% and 6.39 ± 0.13%, respectively. The TSS of GN/CM was significantly (*p* < 0.05) elevated to 7.41 ± 0.17%, suggesting that the incorporation of CM elevated the freshness retention capacity of the coating and delayed the nutrient loss of the strawberries. The LS-rich coating further significantly (*p* < 0.05) increased the TSS, in which the TSS of GN/CM/LS2 was still as high as 8.86 ± 0.47% (highest of all groups) on the sixth day. The enhancement of strawberry TSS by the LS-rich coating might be attributed to the fact that *L. lactis* successfully inhibited the depletion of TSS by *B. cinerea* [55]. 

#### 3.9.5. Decay Rate of Strawberries

The deterioration rate of strawberries is a critical determinant of their commercial worth and suitability for consumption. As shown in Figure 6e, on Day 6, the strawberry in the control group had the highest decay rate of 90.28 ± 6.36%, while the GN/CM/LS2 group had the lowest strawberry decay rate of 9.72 ± 2.41%. The significant results indicated that both CM and *L. lactis* significantly (*p* < 0.05) improved the ability of GN-based coating to preserve strawberries. The improvement could be due to the enhanced barrier ability of the coating matrix induced by CM, reducing the exchange of materials between strawberries and the external environment [24]. Meanwhile, the antifungal substances produced by *L. lactis* inhibited the propagation and metabolism of strawberry pathogenic fungi, thus delaying the decay of strawberries. The same outcomes were observed by Li et al. [56]. They found that sodium alginate/konjac glucomannan/carboxymethylcellulose sodium composite coatings doped with *Cryptococcus albidus* and *Candida parapsilosis* were effective in reducing the decay rate of snap beans. The decay rate decreased significantly (*p* < 0.05) with increasing *L. lactis* concentration, but the change was not significant (*p* > 0.05) between 2% and 4%.

## 4. Conclusions

In summary, an *L. lactis* and CM-loaded GN-based coating with improved physical and antifungal properties was successfully prepared in this study. The incorporated *L. lactis* obviously enhanced the antifungal properties of the GN coating and the 2.0% *L. lactis* achieved the best effect. CM could not only used as a protective agent to improve the viability of probiotics in the GN coating but also as a functional additive to enhance the flexibility and water and oxygen barrier properties of the coating. The *L. lactis* and CM-loaded coating also improved the postharvest quality of strawberries. Based on the results of physical, biological, and preservation performance, GN/CM coatings with 2% to 4% LS could effectively improve the storage quality of strawberries. GN/CM/LS coatings could be used as a novel active coating for strawberry preservation.

## Figures and Tables

**Table 1:** Mechanical and barrier properties, colour, and *MC* of the GN, GN/CM, GN/CM/LS1, GN/CM/LS2, and GN/CM/LS4 films.

Sample	*TS* (MPa)	*EAB* (%)	L*	a*	b*	Δ*E*	*WVP* (kg·m·m^−2^·pa^−1^·s^−1^)	*MC* (%)
GN	7.86 ± 0.386 ^a^	27.47 ± 0.556 ^b^	94.017 ± 1.116 ^a^	−0.3967 ± 0.0231 ^a^	1.8867 ± 0.1097 ^a^	23.33 ± 0.624 ^a^	(7.017 ± 0.808) ^a^ × 10^−13^	15.783 ± 0.294 ^d^
GN/CM	6.4433 ± 0.11 ^b^	39.167 ± 0.643 ^a^	93.1467 ± 0.0681 ^a^	−0.4067 ± 0.0252 ^a^	1.943 ± 0.198 ^a^	22.853 ± 0.206 ^a^	(4.719 ± 0.533) ^b^ × 10^−13^	13.533 ± 0.356 ^e^
GN/CM/LS1	6.343 ± 0.306 ^b^	39.5 ± 0.693 ^a^	92.93 ± 0.486 ^a^	−0.44 ± 0.0458 ^a^	2.097 ± 0.414 ^a^	22.613 ± 0.573 ^a^	(4.728 ± 0.617) ^b^ × 10^−13^	18.017 ± 0.194 ^c^
GN/CM/LS2	5.9933 ± 0.0379 ^bc^	40.7 ± 1.587 ^a^	92.877 ± 0.461 ^a^	−0.4433 ± 0.0252 ^a^	1.97 ± 0.2 ^a^	22.703 ± 0.381 ^a^	(4.908 ± 0.67) ^b^ × 10^−13^	25.143 ± 0.294 ^b^
GN/CM/LS4	5.6267 ± 0.107 ^c^	40.87 ± 2.94 ^a^	94.05 ± 1.482 ^a^	−0.4533 ± 0.1079 ^a^	1.967 ± 0.479 ^a^	23.3 ± 0.805 ^a^	(7.128 ± 0.375) ^a^ × 10^−13^	29.767 ± 0.244 ^a^

Non-significant (*p* > 0.05) and significant (*p* < 0.05) differences are indicated by the same and different letters, respectively. *TS*: tensile strength; *EAB*: elongation at break; *WVP*: water vapour permeability; *MC*: moisture content.

## Data Availability

The original contributions presented in the study are included in the article and the Supplementary Materials, further inquiries can be directed to the corresponding authors.

## Supplementary Materials

The following supporting information can be downloaded at: https://www.mdpi.com/article/10.3390/foods13071102/s1, Figure S1. The number of viable cells of *L. lactis* in films. Non-significant (*p* > 0.05) and significant (*p* < 0.05) differences are indicated by the same and different letters, respectively. GN: gelatin; CM: chia mucilage; LS: *Lactococcus lactis*.

## Abbreviations

GN: gelatin; CM: chia mucilage; LS: Lactococcus lactis; *WVP*: water vapour permeability; *EAB*: elongation at break; *TS*: tensile strength; TSS: total soluble solids; FTIR: Fourier transform infrared; *MC*: moisture content; SEM: scanning electron microscope; *OP*: oxygen permeability; ASTM: American Society of Testing Materials.
