# Supplementary material for: Incorporation of Lactococcus lactis and Chia Mucilage for Improving the Physical and Biological Properties of Gelatin-Based Coating: Application for Strawberry Preservation
Source: Foods. 2024 Apr 3;13(7):1102. doi: 10.3390/foods13071102 (PMC11011328; doi:10.3390/foods13071102)
Supplement: Supplementary file 1 [file foods-13-01102-s001.zip › foods-2905660-supplementary.pdf]

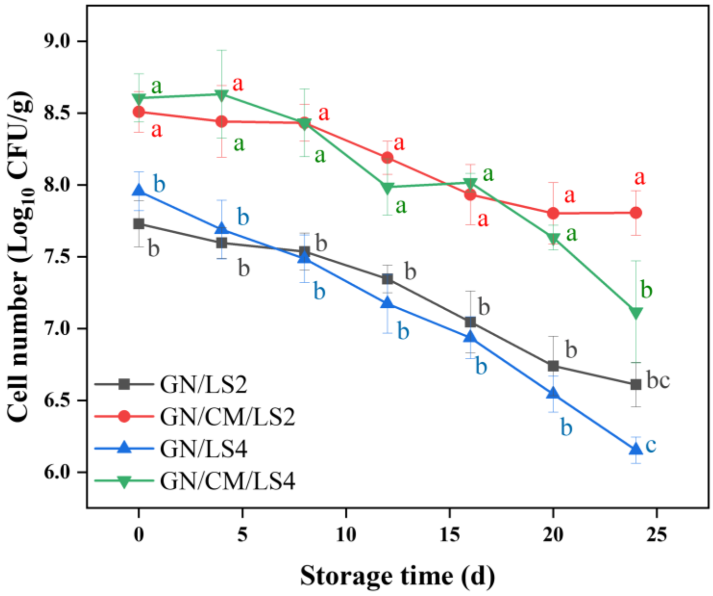

Figure S1. The number of viable cells of *L. lactis* in films. Non-significant (P >0.05) and significant (P <0.05) differences are indicated by the same and different letters, respectively. GN: gelatin; CM: chia mucilage; LS: *Lactococcus lactis*.
